# Mir-1, miR-122, miR-132, and miR-133 Are Related to Subclinical Aortic Atherosclerosis Associated with Metabolic Syndrome

**DOI:** 10.3390/ijerph18041483

**Published:** 2021-02-04

**Authors:** Agnė Šatrauskienė, Rokas Navickas, Aleksandras Laucevičius, Tomas Krilavičius, Rūta Užupytė, Monika Zdanytė, Ligita Ryliškytė, Agnė Jucevičienė, Paul Holvoet

**Affiliations:** 1 Clinic of Cardiac and Vascular Diseases, Institute of Clinical Medicine, Faculty of Medicine, Vilnius University, 08661 Vilnius, Lithuania; agne.satrauskiene@santa.lt (A.Š.); aleksandras.laucevicius@mf.vu.lt (A.L.); ligita.ryliskyte@santa.lt (L.R.); juceviciene.agne@gmail.com (A.J.); 2 Centre of Cardiology and Angiology, Vilnius University Hospital, Santaros Klinikos, 08410 Vilnius, Lithuania; 3 Experimental, Preventive, and Clinic Medicine Department, Centre for Innovative Medicine, 08406 Vilnius, Lithuania; 4 Informatics Faculty, Vytautas Magnus University, 44248 Kaunas, Lithuania; t.krilavicius@bpti.lt (T.K.); ruta.uzupyte@bpti.lt (R.U.); 5 Baltic Institute of Advanced Technology, 01124 Vilnius, Lithuania; 6 Faculty of Mathematics and Informatics, Vilnius University, 03225 Vilnius, Lithuania; 7 Department of Cardiology and Cardiovascular Medicine, Universität Tübingen, 72074 Tübingen, Germany; monika.zdanyte@med.uni-tuebingen.de; 8 Experimental Cardiology, Department of Cardiovascular Sciences, KU Leuven, 3000 Leuven, Belgium; paul.holvoet@kuleuven.be

**Keywords:** subclinical atherosclerosis, arterial markers, metabolic syndrome, miRs, diagnosis

## Abstract

Previously, miR-1, miR-122, miR-126, miR-132, miR-133, and miR-370 were found to be related to coronary artery disease (CAD) progression. However, their relationship with subclinical atherosclerosis, especially in subjects with metabolic syndrome, is unknown. Therefore, our aim was to determine their relationship with arterial markers of subclinical atherosclerosis. Metabolic syndrome subjects (n = 182) with high cardiovascular risk but without overt cardiovascular disease (CVD) were recruited from the Lithuanian High Cardiovascular Risk (LitHiR) primary prevention program. The ardio-ankle vascular index (CAVI), augmentation index normalized to a heart rate of 75 bpm (AIxHR75), aortic pulse wave velocity (AoPWV), and carotid artery stiffness were assessed. MicroRNAs (miRs) were analyzed in serum. Pearson correlation and a univariate linear regression *t*-test showed that miR-1, miR-133b, and miR-133a were negatively associated with CAVI mean, whereas miR-122 was positively associated. MiR-1, miR-133b and miR-133a, and miR-145 were negatively associated with AIxHR75. MiR-122 correlated negatively with AoPWV. In multivariate linear regression models, miR-133b and miR-122 predicted CAVImean, miR-133 predicted AIxHR75, and miR-122 predicted AoPWV. MiR-132 predicted right carotid artery stiffness, and miR-1 predicted left carotid artery stiffness. The addition of smoking to miR-133b and miR-122 enhanced the prediction of CAVI. Age and triglycerides enhanced the prediction of AoPWV by miR-122. A cluster of four miRs are related to subclinical atherosclerosis in subjects with metabolic syndrome. Combined, they may have a more substantial diagnostic or prognostic value than any single miR. Future follow-up studies are needed to establish their clinical relevance.

## 1. Introduction

Cardiovascular disease (CVD) remains the leading global cause of death and is estimated to cause more than 23.6 million deaths per year by 2030 [1]. CVD risk assessment, especially primary prevention and early detection of CVD, remains the essential daily clinical practice endeavors. Coronary artery disease (CAD) is the most common type of heart disease. CAD is a major cause of mortality and morbidity all over the world [2]. The main problem in CAD diagnosis is that clinical manifestations occur only late after subclinical atherosclerosis initiation. Indeed, atherosclerosis is a chronic and relatively benign disease, which progresses silently and is asymptomatically present in most middle-aged individuals [3]. 

To reduce the burden of CVDs, they should be either prevented or diagnosed at their earliest stages. At present, patients’ cardiovascular risk is calculated using scoring systems, taking into account the relative importance of risk factors and quantifying the absolute level of CVD risk for individuals without CVD history. A large number of risk scoring systems are based on data from the Framingham study [4,5]. More recently, a combination of risk factors that commonly cluster together has been termed metabolic syndrome (MetS). This cluster contains dyslipidemia, hypertension, and hyperglycemia, with our without obesity, according to the used definition. The vast majority of studies show that atherosclerosis progresses faster in patients with MetS [6,7]. Framingham cohort studies demonstrated that MetS alone predicted approximately 25% of all new onset CVD cases [8].

Another approach to detecting those at high risk is to look for the presence of subclinical atherosclerosis. Arterial markers, such as the cardio-ankle vascular index (CAVI) [9], augmentation index normalized to a heart rate of 75 bpm (AIxHR75) [10], mean arterial pressure (MAP) [11], aortic pulse wave velocity (AoPWV) [12], carotid artery stiffness [13,14], and flow-mediated dilatation (FMD) [15], are already used in daily clinical practice. They proved to be useful in determining the degree of subclinical carotid atherosclerosis or endothelial dysfunction and have the advantage of being non-invasive and safe. However, their applicability in routine risk estimation is limited because they are operator dependent, resource heavy, and requiring relatively expensive equipment. Therefore, there is a need for more straightforward, less time-consuming, and operator-independent biomarkers that would correlate with these arterial markers, substituting the currently used biomarkers.

We aim to introduce microRNAs (miRs) as novel biomarkers for improved risk classification as they are easy to measure, non-invasive, and cost-effective. miRs are endogenous, non-coding, and small (18–22 nucleotides) RNA molecules regulating gene expression at the post-transcriptional level by imperfect base pairing with the 3′- and 5′ untranslated region (UTR) of target mRNA, leading to mRNA degradation or translation repression [16]. Certain miRs in plasma or serum were proved to be elevated or decreased (already) in the early stages of obesity, MetS, Type 2 diabetes mellitus, hypertension, dyslipidemia, coronary artery disease, peripheral artery disease, heart failure, and acute coronary syndrome [17,18,19,20,21,22,23,24,25,26]. Our recent systematic review [27] showed that miR-1 and miR-145b are potential biomarkers of acute coronary syndromes (ACS). miR-1 has higher sensitivity for all acute myocardial infarction (AMI), and miR-145 has higher sensitivity for ST-elevation myocardial infarction (STEMI) and the worst AMI outcome. However, when miRs were studied across different ACS study populations, patients had varying degrees of coronary stenosis, an essential confounder that limited the ability to pool the study results quantitatively. In addition to miR-1 and miR-133, our systemic review identified miR-122, miR-126, miR-132, miR-133, and miR-370 to be related to disease progression when comparing non-CAD vs. CAD. Therefore, we aimed to study the association of these miRs with several arterial atherosclerosis markers, as described above. Because MetS is associated with increased cardiovascular risk [28,29,30], and information about miRs that predicts subclinical atherosclerosis in the background of MetS is even rarer, we mainly searched correlations between miRs and arterial markers in subjects with MetS, without any previously diagnosed CVD.

## 2. Materials and Methods 

Patients included in our study were recruited between 2007 and 2014 from the Lithuanian High Cardiovascular Risk (LitHiR) primary prevention program [31]. This long-term program has focused on employable-aged women (aged 50–65) and men (aged 40–55) without overt CVD. CVD was defined as stable angina pectoris, coronary artery disease, acute myocardial infarction, coronary artery bypass grafting, percutaneous coronary intervention, transient ischemic attack or stroke, and peripheral artery disease. As part of the program, a two-level approach involving primary healthcare institutions (PHCIs) and specialized cardiovascular prevention units (CVPUs) was applied. Participants of the first level of the program were recruited in three ways. The first group consisted of subjects registered in PHCIs and invited by general practitioners to participate. The second group consisted of subjects who visited PHCIs for reasons other than cardiovascular problems. The third group included people who found out about the program via local mass media. The only participants matching the program criteria were recruited. After cardiovascular risk evaluation at the PHCI level, subjects with high cardiovascular risk were directed to the CVPUs (secondary level) for further examination and treatment. Patients at high risk had MetS with at least one or more of the following conditions: (1) Systematic Coronary Risk Evaluation (SCORE) [32] score of ≥11; (2) type 2 diabetes; (3) positive family history of cardiovascular disease; and/or (4) severe dyslipidemia. 

### 2.1. Selecting Patients with Metabolic Syndrome

From 2006 to 2010, 266,391 patients were examined at the PHCIs. Among them, 3194 (1152 (36.1%) men and 2042 (63.9%) women) subjects were diagnosed with MetS, according to ATP III [33], and referred to the CVPU at the Vilnius University Hospital Santaros Klinikos for additional assessment, risk stratification, and creating an individual prevention plan. All participants gave written informed consent. Out of the 3194 initially screened patients with MetS, blood samples were taken. Patients were reevaluated after a minimum of 3 years of follow-up. Blood samples were taken from 2891 patients after a 12-h fast. We only included the first 178 patients due to limited human and financial recourses in our prospective study.

### 2.2. Measurements of Arterial Markers

Measurements of arterial markers were made during each assessment (every few years, when the patient was screened). Therefore, all patients would have at least two measurements in time (with ±3 years interval). PWV measurements were taken with a Sphygmacor device. The reading was registered when at least 10 consecutive heartbeats with pulse waves matching each other, with at least 95% accuracy, were recorded.

### 2.3. Sample Collection and miR Detection

Venous blood samples (3 EDTA tubes, 2 tubes containing clot activator and gel for serum separation, 1 tube containing sodium citrate and liquid density medium) were collected from MetS patients after a 12-h fast. All blood samples were transported to the clinical laboratory within 30 min, under room temperature. If the sample did not arrive within 30 min after collection, it was not registered and not analyzed. The register contained each sample with its position in the freezer, identified by a code. Registered and coded samples were delivered to the sample preparation room.

### 2.4. Total Blood Cell Preparation

EDTA blood was diluted in erythrocyte lysis (EL) buffer with ratio 1:5, incubated on ice for 10–15 min, and centrifuged (400× *g* for 10 min, 4 °C). After withdrawing the supernatant, the pellet was resuspended in EL buffer (1 volume of blood mixed with 2 volumes EL buffer) and centrifuged (400× *g* for 10 min, 4 °C). After withdrawing the supernatant, pellet blood cells were stored at −80 °C.

### 2.5. Serum Sample Preparation

Blood with clot activators was placed in a vertical position for 30 min and centrifuged (1100–1300× *g*, 10–15 min). The supernatant containing serum was stored under −80 °C. Plasma and serum miRs were selected for the study because they are recognized as the most suitable clinical application source, as sample collection is quick and quite well reproducible [34,35,36].

### 2.6. Sample Testing

miRs were isolated from 200 μL serum using the miRCURY™ RNA Isolation Kit –Biofluids (Exiqon A/S, Vedbaek, Denmark), according to the manufacturer’s instructions. To compare the yield of RNA obtained from serum samples, bacteriophage MS2 carrier RNA was added to each sample during the purification procedure. Reverse transcription (RT) was performed using the Universal cDNA Synthesis Kit II (Exiqon A/S) and the miRCURY LNA Universal RT microRNA PCR, according to the manufacturer’s instructions. Before using RNA samples for miR profiling, the yield of typical serum miRs, absence of polymerase chain reaction (PCR) inhibitors, and hemolysis in the samples was assessed by use of a miR QC PCR Panel (Exiqon A/S). The amplification was performed in a LightCycler 480 Real-Time PCR System (Roche) in 96-well plates with 45 amplification cycles employing the cycling parameters recommended by Exiqon. The amplification curves were analyzed using the Roche LC software to determine the quantification cycle (by the second derivative method) and for melting curve analysis. NormFinder and geNorm reference gene validation software (GenEx Version 6, MultiD Analyses) were used to identify the most stably expressed miRs. Target miRs were normalized with two internal housekeeping control miRs, including hsa-miR-93-5p and hsa-miR-191-5p, which were identified as the most suitable reference miRs [37,38,39,40,41,42,43]. All data were pre-processed in GenEx Enterprise software (MultiD Analyses) to obtain ΔCq values: ΔCq = (Cq target miRNA − mean Cq of the two reference miRs).

### 2.7. Statistical Methods

The primary data (baseline characteristics) was processed with the Excel 2013 (Microsoft Corporation, Redmond, Washington, USA) program. Further data was processed with the statistical software “R” (R Foundation for Statistical Computing, Vienna, Austria). Continuous variables in two groups were compared with a *t*-test, whereas categorical variables were compared with the Chi-square test with Yates’ correction. To measure the linear dependence between miR and medical tests, we used the parametric Pearson correlation coefficient. To examine whether the linear relationship exists between continuous and categorical variables, we performed a simple linear regression and a *t*-test on the regression line’s slope. Additionally, binary logistic regression and evaluation of performance were performed. In order to evaluate the performance of linear regression models, we used two different measures: mean absolute error (MAE), i.e., the average difference between the predicted value and real observation, and mean absolute percentage error (MAPE), i.e., the average value of the unsigned percentage error [44]. Cut off value for carotid-femoral pulse wave velocity was determined at 9.6 m/s per the established practice of clinical norms based on the expert consensus (expert consensus document on the measurement of aortic stiffness in daily practice using carotid-femoral pulse wave velocity). For the formation of different schemes (models), stepwise regression was used. The results are considered to be statistically significant when *p* < 0.05 [44,45,46,47].

## 3. Results

Table 1 summarizes comparative baseline characteristics of two groups: the larger group (n = 3194), including all patients who were diagnosed with MetS in 2007–2014 participating in LitHiR, and the second group of patients (n = 178) randomly selected from the first group to perform miR testing. Components of MetS were defined according to ATP III: waist circumference, triglycerides, high-density lipoprotein cholesterol, blood pressure, and fasting plasma glucose.

The table shows that groups did not differ according to age, sex, smoking, or number of metabolic syndrome components. Waist circumference was not different, although the miR testing group’s body mass index (BMI) was somewhat lower. Lipid levels and blood pressure do not differ between groups. However, patients in the miR testing group tended to have hypertension less often (92% vs. 97%) and type 2 diabetes less often (9% vs. 20%). Positive family history of cardiovascular diseases was similar in both groups. Hs-C-reactive protein levels were somewhat higher in the miR testing group than in the whole cohort. Finally, the miR testing group was treated often with statins (4.9% vs. 16.9 %). 

Table 2 shows different plasma levels of 10 selected miRs in the group of 178 MetS patients.

Pearson correlation (if both variables are continuous) or a linear regression analysis (in case one variable is continuous and another is categorical) were performed to determine the linear relationship between miRs and selected arterial markers (Table 3).

miR-1, miR-133b, and miR-133a were negatively associated with CAVI mean, whereas miR-122 was positively associated. MmiR-1, miR-133b and miR-133a, and miR-145 were also negatively associated with AIxHR75. miR-122 correlated negatively with AoPWV. None of the tested miRs correlated with MAP or FMD%. miR-1 was weakly associated with left carotid artery stiffness, whereas miR-132 was weakly associated with right carotid artery stiffness; none of them were associated with mean carotid artery stiffness in both carotid arteries. miR-126, miR-155, miR-195, and miR-370 were not associated with any of the arterial markers. miR-132 correlated with a plaque in the left carotid artery, whereas miR-370 correlated with a plaque in the right carotid artery. 

Multivariate linear regression models predicting the association between miRs and the investigated arterial markers were built, including only miRs significantly related in univariate models. miR-133b and miR-122 predicted CAVImean. miR-133 predicted AIxHR75, and miR-122 predicted AoPWV. Finally, miR-132 predicted right carotid artery stiffness and miR-1 predicted left carotid artery stiffness (Table 4).

We then investigated whether the addition of other cardiovascular risk markers than miRs could improve the models. The only factors significantly related to arterial markers were incorporated in the model. In model 1, age, sex, and smoking were added to miR expressions. In model 2, low-density lipoprotein cholesterol (LDL-Ch), high-density lipoprotein cholesterol (HDL-Ch), and triglycerides (TG) concentrations were added to model 1. In model 3, C-reactive protein (hs-CRP) values were added to model 2. In model 4, diabetes mellitus diagnosis (presence/absence) was added. 

The addition of age and smoking to miR-133b and miR-122 enhanced the prediction of CAVI (Table 5).

None of the additional factors risk factors improved the prediction of AixHR75 by miR-133b (Table 6).

Age and TG added to the prediction of AoPWV by miR-122 (Table 7). 

None of the other risk factors added to the prediction of carotid artery stiffness by miR-1 or miR-132 (Table 8).

## 4. Discussion

Our study identified four miRs correlating with several subclinical atherosclerosis markers in patients with metabolic syndrome: miR-1, miR-122, miR-132, and miR-133. miR-122, miR-132, and miR-133 were related to markers of arterial stiffness. miR-1 and miR-132 were rather weakly related to carotid stiffness. In contrast, miR-126, miR-145, miR-155, and miR-195 were not related to any arterial markers. Thus, this study further supports the conclusions of our systematic review that miR-133 and miR-1, which belong to the same transcriptional unit, are among the most promising miR biomarkers for progressing stages of cardiovascular disease. 

However, in contrast to the conclusion of our systematic review of published studies that miR-1 is specific for acute coronary syndromes, our current study shows that it is already decreased in patients with asymptomatic atherosclerosis. The relation of the selected miRs with arterial markers of atherosclerosis has been previously underscored by mechanistic data supporting their involvement in several pathogenic mechanisms in atherosclerosis. Indeed, they were found to regulate endothelial function and angiogenesis (miR-1, miR-133) [47,48], vascular smooth muscle cell differentiation (miR-133) [49], apoptosis (miR-1 and miR-133) [50,51,52], inflammation (miR-133) [52,53,54,55], cardiac myocyte differentiation (miR-1 and miR-133) [50,56], and cardiac fibrosis (miR-122 and miR-133) [57,58,59,60]. In addition, our current study [27] adds two other miRs: miR-122 and miR-132. They were both related to aortic stiffness. miR-122 is associated with cardiac fibrosis [61]. miR-122 is one of a series of miRs, which may play a mechanistic role in coupling lipid metabolism and atherosclerosis [61]. miR-122 may reduce nitric oxide levels and induce endothelial dysfunction, particularly in hypertension [62,63]. Additionally, miR-122 down-regulates miR-21, enhancing cholesterol synthesis by increasing HMGCR (3-hydroxy-3-methylglutaryl-co-enzyme A reductase) transcription and translation [64,65]. Additionally, it has been implicated in inflammation in general [66]. Wang et al. found out that miR-122 is associated with obesity and insulin resistance in young adults [67]. Gao et al. concluded that miR-122 and miR-370 are higher in patients with hyperlipidemia and associated with CAD [21]. miR-122 was also associated with glycemic status and insulin levels. It was significantly upregulated in individuals with type 2 diabetes mellitus, obesity, and in the pre-diabetic stage [68]. Interestingly, TG and smoking added to the predictive power of miR-122. miR-132 has been implicated in smooth muscle cell proliferation in intimal hyperplasia [69] and vascular endothelial inflammation [70]. Eskildsen et al. found that miR-132 is involved in angiotensin II-induced hypertension [71]. Altogether, the relation of miR-122 and miR-132 with inflammation may explain their additive value to the expected values of miR-1 and miR-133 as biomarkers. 

Overall, our study adds to earlier studies, showing that a panel of miRs embodies the intricate mechanisms in different plaque progression stages [72]. Further, Ren et al. identified a cluster of miRs (miR-106b/25 cluster, miR-17/92a cluster, miR-21/590-5p family, miR-126* miR-451) to be dysregulated in patients with vulnerable CAD compared with controls [73]. D’Alessandra et al. demonstrated that miR-1, miR-133a, and miR-126 in unstable CAD and miR-1, miR-126, and miR-485-3p in stable CAD correctly classified patients vs. controls with an efficiency of 87% [74]. Previous studies revealed the importance of miRs in regulating critical signaling and lipid homeostasis pathways that alter the balance of atherosclerotic plaque progression and regression [61,75,76]. In brief, the identified miRs are related to many other atherogenic processes, underscoring their mechanistic and clinical relevance.

A recent review discussed other commonly used subclinical vascular markers in the clinical setting. Among them were the carotid intima-media thickness and plaque measured by ultrasound, coronary artery calcium detected by cardiac computed tomography, ankle–arm index pressure measured by distal pressure Doppler measurement, and aortic pulse wave velocity measured from carotid and femoral pressure wave recordings with a Doppler or mechanographic device. Positive testing for subclinical atherosclerosis was found to be associated with a moderately high to high coronary heart disease (CHD) risk. However, the different types of subclinical atherosclerosis tests had a different prognostic performance. Positive testing for intima-media thickness, ankle-arm index pressure, and aortic pulse wave velocity conveyed a 10-year CHD risk between 10% and 20%. In contrast, positive testing for carotid plaque or coronary calcium conveys a 10-year CHD risk superior to 20%. In contrast, negative testing for subclinical atherosclerosis conveyed a low CHD risk inferior to 10%, whatever the test considered. It was concluded that for individuals at intermediate risk, e.g., 10–20% 10-year Framingham risk of fatal and non-fatal CHD or 3–5% European SCORE risk of fatal cardiovascular disease, clinicians may consider testing for subclinical atherosclerosis, and, in those with a positive test, aggressive risk reduction intervention may be appropriate [77,78,79,80,81,82,83]. CAVI was found to increase linearly with age and was elevated even in mild arteriosclerotic disease. It could identify differences in the degree of arteriosclerosis among patients with severe arteriosclerotic disease and better reflected the severity of disease of the coronary artery than brachial-ankle pulse wave velocity. Patients with higher CAVI values showed a poor prognosis compared with those with lower CAVI values. Furthermore, CAVI could be lowered by controlling hyperglycemia and hypertension [84], essential factors of MetS. Given this data, the relation of miR-133b and miR-122 with CAVI is of interest. Nevertheless, the implementation of subclinical atherosclerosis testing in patients’ risk management is still considered dependent on a better knowledge of the comparative prognostic performance of various atherosclerosis tests currently available.

Surprisingly, miR-126, miR-45, and miR-155 were not associated with any arterial markers in our study. However, together with miR-30a and let-7, miR-126 may be more linked to ischemic cardiovascular disease, including strokes [85,86]. Exosomes enriched in miR-126 reduced ischemic injury and fibrosis in a preclinical model [87]. The relation of miR-126 expression with obesity and type 2 diabetes remains to be elucidated. Indeed, one study showed that miR-126 is significantly lower (~65%) in overweight and obese subjects [88]. Another study showed that miR-126 was up-regulated in obesity and down-regulated in the pre-diabetic stage and in type 2 diabetes mellitus [89].

Previously, low levels of circulating miR-145 and miR-155 were found to be associated with CAD, by comparing CAD patients with healthy controls [90]. However, it is possible that this relation was due to differences in inflammation. Obesity-induced inflammation is an important mechanism linking obesity to MetS, and miR-145 stimulates the expression of TNF-α in adipocytes [91]. Additionally, miR-155 increases leukocyte-endothelial cell interactions and endothelial cell migration [92] and amplifies inflammation in adipocytes [93]. miR-145 may be more related to acute coronary artery disease. Indeed, miR-145 was lower in AMI patients, through was in relation to TGF-β- and hypoxia-induced ischemia [86,94]. miR-155 expression correlated positively with the Gensini score [95]. The up-regulation of miR-155 by hypertension and inflammation is associated with endothelial dysfunction and loss of protection against ox-LDL-induced cytokine release and apoptosis [96,97,98,99,100]. Circulating miR-195 predicted adverse ischemic events after angioplasty with stent implantation [101], possibly preventing endothelial repair [102]. Thus, differences in the regulation of certain miRs according to disease stages may explain why particular miRs do not show a significant association with arterial markers in our study.

Our data is promising and suggests new means of biomarker-based risk stratification for subclinical atherosclerosis. However, the miR group’s relatively small size, the differences between the miR group and the whole cohort, and the lack of follow-up prevent establishing firm conclusions about their diagnostic power. The lack of data on plaque calcification is a further limitation. Notwithstanding these limitations, our study is unique because several markers of subclinical atherosclerosis have been included.

## 5. Conclusions

In conclusion, our study identified a cluster of four miRs (miR-1, miR-122, miR-132, and miR-133) related to subclinical atherosclerosis in MetS patients. Their value is underscored by published mechanistic data, implicating them in several atherogenic processes, such as endothelial dysfunction and inflammation, angiogenesis, vascular smooth muscle cell proliferation and differentiation, and apoptosis, hallmarks of early atherosclerosis. Further studies define the association between miRs and MetS as a set of components, and validation of those findings in a non-MetS population might be valuable. 

## Figures and Tables

**Table 1:** Baseline characteristics.

Baseline Characteristics.	miR	All Patients	*p*-Value
Number of patients	178	3194	
**Sex**			
Male n (%)	73 (41)	1152 (36.1)	0.210
Female n (%)	105 (59)	2042 (63.9)	
Age, years (mean ± SD)	53.23 ± 5.69	54.12 ± 6.15	0.109
**MetS components**			
Waist circumference men (mean ± SD)	109.4 ± 7.02	110.89 ± 9.88	0.102
Waist circumference women (mean ± SD)	102.9 ± 9.1	104.62 ± 10.65	0.142
Body mass index (mean ± SD)	31.25 ± 3.94	32.33 ± 4.83	0.026
Triglycerides (mean ± SD)	2.63 ± 4.14	2.58 ± 2.42	0.489
High-density lipoprotein cholesterol (mean ± SD)	1.26 ± 0.28	1.24 ± 0.33	0.204
Systolic blood pressure, mmHg (mean ± SD)	135.64 ± 10.81	142.04 ± 16.85	0.143
Diastolic blood pressure, mmHg (mean ± SD)	83.18 ± 8.88	86.79 ± 10.58	0.104
Fasting plasma glucose, mmol/L (mean ± SD)	5.9 ± 0.72	6.31 ± 1.36	<0.001
**Number of MetS components**			**0.225**
3 out of 5 n (%)	74 (41.6)	1197 (37.5)	0.225
4 out of 5 n (%)	60 (33.7)	1147 (35.9)	0.225
5 out of 5 n (%)	33 (18.5)	767 (23.1)	0.225
**Mean number of MetS components**	3.64 ± 0.87	3.85 ± 0.78	0.225
**Other cardiovascular risk factors**			
Arterial hypertension (%)	164 (92.1)	3089 (96.7)	0.003
Hyperlipidemia (%)	178 (100)	3157 (98.8)	0.283
Positive family history (%)	68 (38.2)	1031 (32.3)	0.119
Diabetes mellitus (%)	16 (9)	644 (20.2)	0.0004
Current smoking (%)	43 (24.2)	734 (22.9)	0.786
C-reactive protein (mean ± SD)	3.5 ± 4.58	2.93 ± 3.67	0.003
Low-density lipoprotein cholesterol (mean ± SD)	4.44 ± 1.26	4.34 ± 1.26	0.203
Use of statins	30 (16.9)	158 (4.9)	<0.001

Metabolic syndrome (MetS), microRNA (miR).

**Table 2:** Plasma levels of miRs.

	miR-1	miR-126	miR-145	miR-155	miR-122	miR-370	miR-133a	miR-133b	miR-195	miR-132
**Mean ± SD**	6.12 ± 1.33	2.05 ± 0.6	2.11 ± 0.67	5.79 ± 1.1	−0.92 ± 1.3	7.98 ± 1.71	6.05 ± 1.18	4.58 ± 1.2	7.09 ± 1.43	4.66 ± 0.98

microRNA (miR), standard deviation (SD)

**Table 3:** Pearson correlation.

		CAVI Mean	AIxHR75	AoPWV	MAP	FMD %	Stiffness Left Carotid Artery	Stiffness Right Carotid Artery	Stiffness Carotid Artery	Right Carotid Plaque	Left Carotid Plaque
**miR-1**	***r***	**−0.218**	**−0.201**	3.7 × 10^−5^	−0.076	−0.086	**0.154**	−0.024	0.074	−0.05	0.027
	***p***	**0.004**	**0.005**	0.99	0.315	0.264	**0.042**	0.754	0.324	0.63	0.79
**miR-133b**	***r***	**−0.221**	**−0.242**	−0.09	−0.066	−0.073	0.109	0.024	0.076	−0.019	−0.021
	***p***	**0.003**	**0.001**	0.233	0.385	0.347	0.151	0.757	0.318	0.855	0.835
**miR-133a**	***r***	**−0.177**	**−0.235**	−0.003	−0.071	−0.067	0.053	0.014	0.039	0.039	−0.038
	***p***	**0.02**	**0.002**	0.964	0.345	0.389	0.482	0.858	0.613	0.712	0.699
**miR-122**	***r***	**0.152**	0.032	**−0.15**	−0.1	0.002	0.036	0.102	0.078	−0.121	−0.065
	***p***	**0.046**	0.671	**0.049**	0.188	0.98	0.639	0.181	0.306	0.244	0.512
**miR-145**	***r***	0.037	**−0.151**	0.005	−0.039	−0.066	0.001	0.038	0.022	−0.025	−0.033
	***p***	0.629	**0.044**	0.944	0.603	0.392	0.986	0.615	0.769	0.811	0.74
**miR-132**	***r***	0.025	0.008	0.057	0.011	0.091	0.018	**0.165**	0.103	0.118	**0.176**
	***p***	0.747	0.92	0.453	0.889	0.24	0.818	**0.029**	0.174	0.257	**0.04**
**miR-126**	***r***	−0.054	−0.041	−0.003	−0.009	−0.03	−0.002	0.119	0.067	0.091	0.102
	***p***	0.479	0.585	0.972	0.907	0.695	0.985	0.116	0.382	0.382	0.306
**miR-155**	***r***	−0.136	−0.126	−0.064	0.041	−0.074	−0.035	−0.032	−0.038	0.006	-0.021
	***p***	0.073	0.095	0.393	0.589	0.34	0.642	0.679	0.616	0.953	0.834
**miR-195**	***r***	0.065	−0.012	0.075	−0.124	−0.068	0.047	0.103	0.085	−0.169	−0.061
	***p***	0.398	0.873	0.317	0.101	0.382	0.536	0.173	0.261	0.104	0.537
**miR-370**	***r***	−0.058	9 × 10^−4^	0.094	0.068	−0.004	0.026	0.095	0.069	**0.291**	−0.047
	***p***	0.449	0.99	0.213	0.369	0.964	0.729	0.209	0.364	**0.005**	0.636

Augmentation index normalized to a heart rate of 75 bpm (AIxHR75), aortic pulse wave velocity (AoPWV), cardio-ankle vascular index (CAVI), flow-mediated dilatation (FMD), mean arterial pressure (MAP), microRNA (miR). Bolded values—statistically significant associations.

**Table 4:** Linear regression models.

y	Model	MAE	MAPE
CAVImean	9.21 − 0.31 miR-133b + 0.24 miR-122	1.12	17.16
AIxHR75	38.55 − 3.14 miR-133b	9.87	145.89
AoPWV	8.41 − 0.19 miR-122	1.25	14.31
Stiffness right carotid artery	2.31 + 0.39 miR-132	1.35	39.01
Stiffness left carotid artery	3.72 + 0.14 miR-1	1.27	32.69

Augmentation index normalized to a heart rate of 75 bpm (AIxHR75), aortic pulse wave velocity (AoPWV), cardio-ankle vascular index (CAVI), mean absolute error (MAE), mean absolute percentage error (MAPE), microRNA (miR).

**Table 5:** Factors predicting CAVI.

Scheme	Model	MAE	MAPE
1	8.94–0.23 miR-122 − 0.3 **age** miR-133b + 0.82 **smoking**	**1.07**	16.18
2	0.6 + 0.22 miR-122 + 0.12 age + 1.1 smoking − 0.27TG − 0.78 HDL-Ch	1.43	20.07
3	0.6 + 0.22 miR-122 + 0.12 age + 1.1 smoking − 0.27TG − 0.78 HDL-Ch	1.43	20.07
4	0.6 + 0.22 miR-122 + 0.12 age + 1.1 smoking − 0.27TG − 0.78 HDL-Ch	1.43	20.07

Cardio-ankle vascular index (CAVI), high-density lipoprotein cholesterol (HDL-Ch), mean absolute error (MAE), mean absolute percentage error (MAPE), microRNA (miR), Triglycerides (TG). Bolded—significant relation.

**Table 6:** Factor predicting AIxHR75.

Scheme	Model	MAE	MAPE
1	37.49 − 2.06 miR-133b − 10.73 sex	10.58	151.72
2	37.49 − 2.06 miR-133b − 10.73 sex	10.58	151.72
3	37.49 − 2.06 miR-133b − 10.73 sex	10.58	151.72
4	37.49 − 2.06 miR-133b − 10.73 sex	10.58	151.72

Augmentation index normalized to a heart rate of 75 bpm (AIxHR75), mean absolute error (MAE), mean absolute percentage error (MAPE), microRNA (miR).

**Table 7:** Factors predicting AoPWV.

Scheme	Model	MAE	MAPE
1	3.34–0.27 miR-122 + 0.09 age	1.29	14.7
2	3.34–0.26 miR-122 + 0.08 **age** + 0.63 **TG**	**1.22**	13.53
3	3.34–0.26 miR-122 + 0.08 age + 0.63 TG	1.22	13.53
4	3.34–0.26 miR-122 + 0.08 age + 0.63 TG	1.22	13.53

Aortic pulse wave velocity (AoPWV), mean absolute error (MAE), mean absolute percentage error (MAPE), microRNA (miR), Triglycerides (TG). Bolded—significant relation.

**Table 8:** Factors predicting carotid artery stiffness.

**Stiffness right carotid artery**			
**Scheme**	**Model**	**MAE**	**MAPE**
1	−1.92 + 0.4 miR-132 + 0.07 age	1.37	38.07
2	−1.92 + 0.4 miR-132 + 0.07 age	1.37	38.07
3	−2.31 + 0.43 miR-132 + 0.08 age + 0.06 hs-CRP	1.41	38.03
4	−2.31 + 0.43 miR-132 + 0.08 age + 0.06 hs-CRP	1.41	38.03
**Stiffness left carotid artery**			
**Scheme**	**Model**	**MAE**	**MAPE**
1	−0.46 + 0.19 miR-1 + 0.07 age−0.02 smoking duration	1.29	31.73
2	−0.46 + 0.19 miR-1 + 0.07 age−0.02 smoking duration	1.29	31.73
3	−0.92 + 0.18miR-1 + 0.08 age−0.03 smoking duration + 0.07 hs-CRP	1.31	30.43
4	−0.92 + 0.18miR-1 + 0.08 age−0.03 smoking duration + 0.07 hs-CRP	1.31	30.43

High sensitivity C-reactive protein (Hs-CRP), mean absolute error (MAE), mean absolute percentage error (MAPE), microRNA (miR).
